# GLP‐1 Responses to a Single Meal Fortified With Oyster Mushroom Powder in Adults With Impaired Glucose Tolerance Depend on the Gut Microbiota Composition Before the Meal

**DOI:** 10.1002/mnfr.70159

**Published:** 2025-06-29

**Authors:** Linda Klümpen, Anna Donkers, Waldemar Seel, Lisa Dicks, Jens Juul Holst, Peter Stehle, Marie‐Christine Simon, Sabine Ellinger

**Affiliations:** ^1^ Institute of Nutritional and Food Science Human Nutrition University of Bonn Bonn Germany; ^2^ Institute of Nutritional and Food Science Nutrition and Microbiota University of Bonn Bonn Germany; ^3^ Department of Biomedical Sciences University of Copenhagen, Faculty of Health and Medical Sciences Copenhagen Denmark; ^4^ The Novo Nordisk Foundation Center for Basic Metabolic Research University of Copenhagen, Faculty of Health and Medical Sciences Copenhagen Denmark; ^5^ Institute of Nutritional and Food Science Nutritional Physiology University of Bonn Bonn Germany

**Keywords:** β‐glucans, gut microbiota composition, meal fortification, oyster mushroom, postprandial GLP‐1 response

## Abstract

Fortification of a single meal with β‐glucan‐rich oyster mushroom (*Pleurotus ostreatus*) powder has been shown to increase the response of glucagon‐like peptide 1 (GLP‐1) and reduce concentrations of non‐esterified fatty acids (NEFAs) in adults with impaired glucose tolerance (IGT). This secondary analysis of a randomized controlled crossover study (DRKS00015244) aimed to determine whether these effects are modulated by baseline gut microbiota composition. A fecal sample was collected once at baseline before consumption of either a *P. ostreatus*‐enriched meal (EN) or a non‐enriched meal (CON). The microbiota was analyzed using 16S rRNA V3–V4 sequencing. An inverse association was observed between alpha diversity and differences in the meal‐induced GLP‐1 response (*p* < 0.05), whereas NEFA responses appeared unaffected. Notably, only participants with lower microbial evenness showed a greater GLP‐1 response after EN versus CON (*p =* 0.012). Additionally, the presence of *Eubacterium ventriosum* group and *Clostridium methylpentosum* group was associated with increased GLP‐1 concentrations following EN (*p <* 0.05). Baseline gut microbiota composition modulates the GLP‐1 response to a single meal fortified with *β*‐glucan‐rich oyster mushroom powder, with differences in GLP‐1 response being more pronounced in individuals whose microbiome is more specialized in fermenting fiber into SCFAs.

## Graphical abstract

This work showed that the GLP‐1 response to a meal enriched with oyster mushroom powder in adults with impaired glucose tolerance is modulated by the gut microbiome. Higher GLP‐1 responses following the enriched meal were associated with lower microbial evenness and a microbiome more specialized in fiber degradation. Thus, individuals with such a microbiome may especially benefit from this measure.

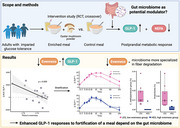

**Abbreviations**
ASV: amplicon sequence variant
AUC: area under the curve
CON: control meal
EN: enriched meal
GLP‐1: glucagon‐like peptide 1
HEG: high evenness group
IGT: impaired glucose tolerance
IQR: interquartile range
LEG: low evenness group
NEFAs: non‐esterified fatty acids
*P. ostreatus*
: *Pleurotus ostreatus*
PD: phylogenetic diversity
PERMANOVA: permutational multivariate analysis of variance
PICRUSt2: phylogenetic investigation of communities by reconstruction of unobserved states 2
RCT: randomized controlled trial
SCFAs: short‐chain fatty acids
sPLS: sparse partial least squares
ΔAUC: baseline‐corrected AUC
Δtime point: baseline‐corrected concentrations at the single postprandial time points

## 1 Introduction

The gut microbiota is a complex and dynamic ecosystem comprising more than a thousand bacterial species, alongside eukaryotes, viruses, and archaea [1]. This diverse microbial community has coevolved with the human host, expanding the human genome by contributing up to 500 times more encoded genes [2]. The composition of the gut microbiota is influenced by a multifaceted interplay of intrinsic factors (e.g., host genetics, age, mode of delivery) and extrinsic factors (e.g., diet, physical activity, geography), resulting in substantial inter‐individual variability [1, 2]. Notably, the gut microbiota is involved in the regulation of host metabolism, and disruptions in microbial balance—referred to as dysbiosis—are associated with various metabolic disorders such as obesity and type 2 diabetes [3].

Several studies have indicated that the gut microbiota influences the host's metabolic responses to dietary intake [2, 4]. For example, the baseline abundance of bacterial genera capable of degrading complex polysaccharides, such as *Prevotella*, has been shown to influence the efficacy of high‐fiber diets in promoting metabolic improvements, including weight loss. These effects are believed to be mediated, at least in part, by appetite regulation through microbially derived SCFAs [5]. Therefore, individual differences in gut microbiota composition may contribute to the wide inter‐individual variability in response to interventions observed across human studies. Thus, characterization of the individual gut microbiota composition may be a useful tool for determining which individuals may benefit the most from a given type and duration of a dietary intervention [6].

In a recently conducted randomized controlled trial (RCT) involving adults with impaired glucose tolerance (IGT), we demonstrated that a single meal fortified with β‐glucan‐rich oyster mushroom powder beneficially affects the plasma levels of glucagon‐like peptide 1 (GLP‐1) and serum concentrations of non‐esterified fatty acids (NEFAs). Our results suggest that fortification of a single meal with oyster mushroom powder may be a suitable measure to improve postprandial metabolism and reduce the risk of obesity in the long‐term due to the satiating properties of GLP‐1 [7]. However, the underlying mechanisms remain unclear.

Given the potential interactions between gut microbiota composition and dietary interventions, we hypothesized that inter‐individual differences in the gut microbiota might influence the metabolic response which was observed after the single consumption of a mushroom powder‐enriched meal. To test this hypothesis, we conducted a secondary analysis to investigate the effect of baseline gut microbiota composition on postprandial biomarker responses.

## 2 Experimental Section

### 2.1 Study Design

An RCT with a double‐blind, crossover design was conducted to examine the metabolic effects of fortifying a single meal with β‐glucan‐rich oyster mushroom powder in 22 adults with IGT. Each participant completed 2 study days, separated by a washout period of at least 1 week. On each study day, following a minimum of 12 h overnight fast, participants consumed either a meal enriched with 20 g powder of oven‐dried *Pleurotus ostreatus* (enriched meal, EN) or a non‐enriched control meal (CON). This amount of mushroom powder (BIO Pleurotus, Wohlrab, Entrischenbrunn, Germany) provided 8.1 g of β‐glucans and was equivalent to a usual serving size of 200 g of fresh *P. ostreatus*.

Venous blood samples were collected before and repeatedly after meal consumption over a 4 h period (0, 15, 30, 45, 60, 90, 120, 180, and 240 min) to assess GLP‐1 and NEFA concentrations. Participants were instructed to maintain their usual lifestyle and body weight throughout the study period, to avoid β‐glucan‐rich foods and alcoholic beverages for 3 days prior to each study day, and to consume a similar dinner the evening before each visit.

Details on the study design, intervention procedures, nutritional composition of the mushroom powder and meal components, physical properties of the meals with and without enrichment, as well as the reproducibility of GLP‐1 and NEFA measurements, have been described previously [7].

### 2.2 Gut Microbiome Sample Processing

A fresh fecal sample was collected once from each participant immediately prior to the first study day and stored at –80°C at the study center. Upon study completion, all samples were transported on dry ice to Life & Brain (Bonn, Germany) for DNA extraction and high throughput 16S rRNA amplicon sequencing. Total genomic DNA was extracted from 120–150 mg of fecal material using the ZymoBIOMICS Mini Kit (Zymo Research Europe, Freiburg, Germany) according to the manufacturer's instructions and stored at −20°C until further analysis. Details on library preparation and 16S metagenome analysis of the fecal microbiome have been reported previously [8]. Briefly, the V3–V4 region of the 16S rRNA gene was amplified using the Bakt_341F and Bakt_805R primer pair. Sequencing was performed on an Illumina MiSeq system (San Diego, CA, USA) using the MiSeq Reagent Kit v3 to generate 2 × 300 bp. Clustering was performed at 4 pM with a 15% PhiX control spike‐in. Reads were demultiplexed on the MiSeq system. Quality control, denoising, and read joining were conducted using QIIME 2 (version 2024.2) with the DADA2 plugin [9, 10].

### 2.3 Gut Microbiome Analysis

Microbiome diversity was calculated in QIIME 2 using a rarefied feature table with a sampling depth of 43,185 sequences, applying default parameters [10]. Alpha diversity metrics included Observed features, Shannon entropy, Pielou's evenness, and Faith's phylogenetic diversity (PD). Beta diversity was assessed using Bray–Curtis, Jaccard, unweighted UniFrac, and weighted UniFrac distance metrics.

For taxonomic analysis, the amplicon sequence variant (ASV) table was aggregated at genus level. The functional potential of the microbiota was predicted using phylogenetic investigation of communities by reconstruction of unobserved states 2 (PICRUSt2, version 2.5.1) [11]. Pathway annotation was performed using the MetaCyc Metabolic Pathway Database (MetaCyc.org, version 23.1) [12]. Features with ≥4 read counts in ≥20% of the samples (prevalence threshold) were retained for downstream analysis. Count data were normalized using centered log‐ratio transformation.

### 2.4 Statistical Analysis

Statistical analyses were performed using SPSS version 29.0 (IBM Corp., Chicago, IL, USA) and R software version 4.3.3 (Boston, MA, USA). The level of statistical significance was set at 5% (two‐tailed), unless otherwise specified.

Postprandial responses for GLP‐1 and NEFAs were quantified as the incremental area under the curve over 4 h (AUC), calculated using the trapezoidal method and excluding the area below (for GLP‐1) or above (for NEFAs) the fasting values (baseline). Baseline‐corrected AUCs (ΔAUC) were calculated using Prism version 10.0 (GraphPad, CA, USA). If a single postprandial value was missing, AUCs for both treatments were calculated excluding that value. However, if more than two consecutive values were missing, the participant was excluded from the analysis.

To determine the impact of gut microbiota diversity on the postprandial response, linear regression analysis was conducted with the alpha diversity index as the independent variable and the difference in the postprandial response between EN and CON (Δ = EN–CON) as the dependent variable. This difference was assessed using (i) the ΔAUC and (ii) the baseline‐corrected concentrations at individual postprandial time points (Δtime point, i.e., changes vs. baseline). Data were standardized by centering (subtracting the mean) and scaling (dividing by the SD). Beta estimates and 95% confidence intervals were reported. Model residuals were visually inspected for normality to assess the validity of statistical assumptions.

To identify bacterial genera and microbial pathways that could explain the difference in the postprandial metabolic response between EN and CON, sparse partial least squares (sPLS) regression analysis was performed using the mixOmics R package [13]. Models were built with leave‐one‐out cross‐validation, and performance was evaluated by the Q^2^ total (cross‐validated R^2^). Additionally, pairwise associations between differences in postprandial metabolic responses and specific genera or microbial pathways were assessed using Spearman's rank correlation.

To further investigate the role of the microbiota composition for the metabolic response, participants were stratified into low and high evenness groups (LEG vs. HEG) based on Pielou's evenness index, using the median value (0.754) as the cutoff. Linear mixed‐effects models with time, treatment, and the time × treatment interaction as fixed effects, and participant ID as a random effect, were used to evaluate the effects of *P. ostreatus* enrichment on the postprandial response within each group. Differences in alpha and beta diversity between LEG and HEG were analyzed using unpaired Student's *t*‐tests and permutational multivariate analysis of variance (PERMANOVA, implemented in the vegan R package [14]), respectively. Differential abundance analysis was performed using MaAsLin2 to identify genera and pathways that differed between the groups [15]. Multiple comparisons were corrected using the Benjamini–Hochberg procedure, and results with *q* values < 0.1 were considered statistically significant.

## 3 Results

### 3.1 Participant Characteristics

A total of 22 participants completed the study per protocol. Six participants were excluded from the secondary analysis for the following reasons: unavailable microbiome data (*n *= 4), missing multiple postprandial blood samples (*n *= 1), or antibiotic therapy within 12 weeks before the first study day (*n *= 1). Consequently, 16 participants were included in the final analysis.

Participants had a median age of 33.5 years (interquartile range [IQR]: 35.0) and a mean BMI of 34.4 ± 6.0 kg/m^2^. The sex distribution was balanced with 50% women and 50% men. Additional details regarding the participants’ metabolic status are provided in Table S1.

### 3.2 Taxonomy of the Gut Microbiome

Based on the total relative abundances in all fecal samples, the 10 most abundant genera were *Blautia* (12.7%), *Bacteroides* (7.8%), *Faecalibacterium* (6.2%), *Bifidobacterium* (5.2%), *Agathobacter* (4.1%), *Subdoligranulum* (3.4%), *Eubacterium hallii* group (3.3%), *Collinsella* (2.8%), *Dorea* (2.5%), and *Akkermansia* (2.4%). Together, these genera comprised approximately 50% of the fecal microbiome, while the remaining 219 genera made up the other 50% (Figure S1A).

### 3.3 Association Between Postprandial GLP‐1 Response and Alpha Diversity

**FIGURE 1:**
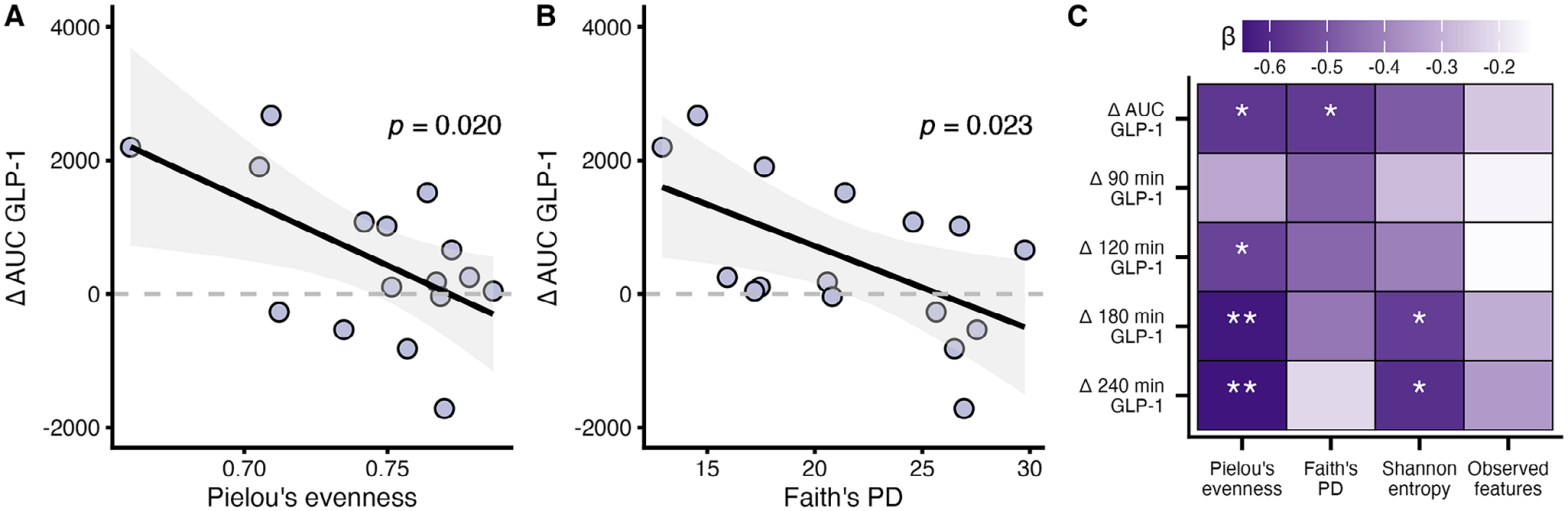
Association between the postprandial GLP‐1 response and alpha diversity. (A) Associations between differences in the meal‐induced GLP‐1 response (∆AUC, pmol L^−1^ min) and Pielou's evenness. (B) Associations between differences in the meal‐induced GLP‐1 response (∆AUC, pmol L^−1^ min) and Faith's PD. (C) Associations between differences in baseline‐corrected GLP‐1 concentrations at later time points between EN and CON (Δtime point, pmol L^−1^) and alpha diversity indices. *p* values were derived from linear regression analysis. * *p < *0.05; ** *p <* 0.01. *n* = 16.

Inverse associations were observed between differences in the meal‐induced GLP‐1 response (∆AUC) and both Pielou's evenness (*p* = 0.020) and Faith's PD (*p* = 0.023) (Figure 1A,B). Furthermore, ∆AUC showed a trend toward an inverse correlation with Shannon entropy (*p *= 0.054), while no association was found with Observed features (*p =* 0.340) (Figure S1B,C, Table S2).

Moreover, inverse associations were observed between the meal‐induced differences in the postprandial GLP‐1 response at later time points and Pielou's evenness (∆120 min to ∆240 min; all *p* < 0.05), as well as with Shannon entropy (∆180 min and ∆240 min; both *p *< 0.05) (Figure 1C, Table S2).

### 3.4 Association Between Postprandial GLP‐1 Response and Bacterial Composition at the Genus Level

**FIGURE 2:**
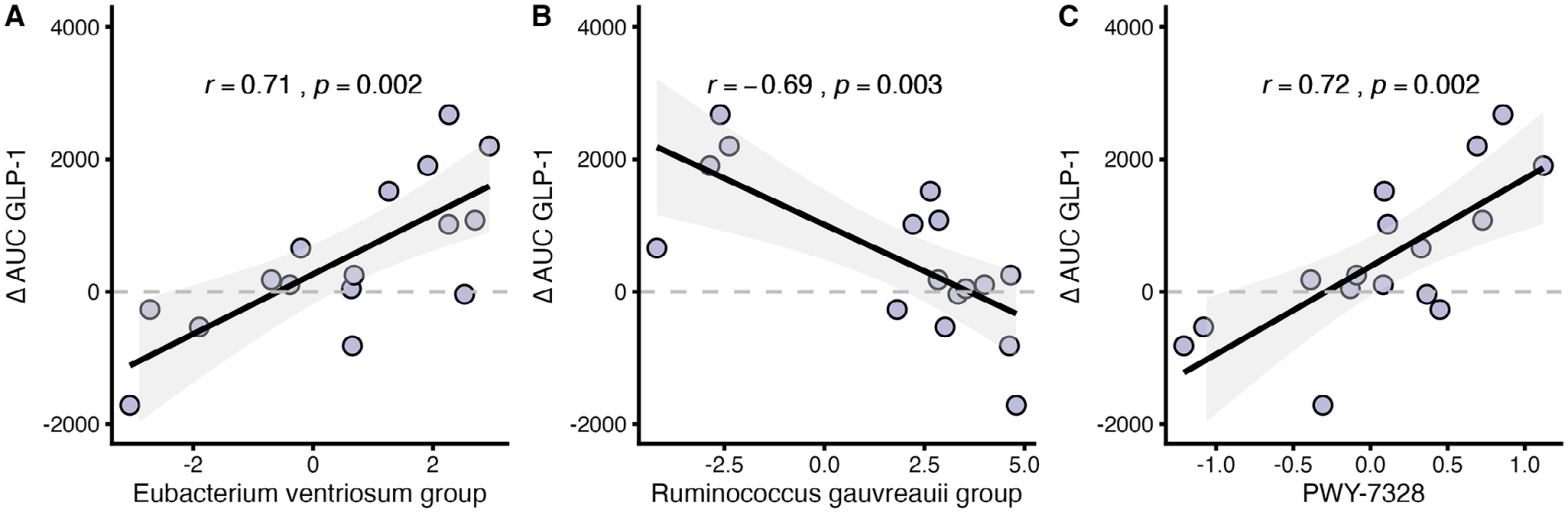
Associations between the postprandial GLP‐1 response and microbial genera as well as functional capacity. Pairwise correlations of (A) the *Eubacterium ventriosum* group, (B) the *Ruminococcus gauvreauii* group, and (C) PWY‐7328 with the differences in the meal‐induced GLP‐1 response (∆AUC, pmol L^−1^ min). *p* values were derived from Spearman's correlation analysis. *n* = 16.

To identify genera that may explain the meal‐induced differences in the postprandial GLP‐1 response (∆AUC), an sPLS regression analysis was conducted (*n* = 138 genera after filtering), identifying the *Eubacterium ventriosum* group and *Ruminococcus gauvreauii* group as the most important taxa (Q^2^ total: 0.433). Additional correlation analyses confirmed that ∆AUC was positively associated with the *E. ventriosum* group (*p = *0.002, Figure 2A) and inversely associated with the *R. gauvreauii* group (*p = *0.003, Figure 2B). Furthermore, these bacterial groups showed consistent positive or inverse correlations with differences in the postprandial GLP‐1 response at all later time points (∆90 min to ∆240 min; all *p* < 0.05) (Table S3).

### 3.5 Association Between Postprandial GLP‐1 Response and Microbial Functional Capacity

To identify microbial pathways that may account for the meal‐induced differences in the postprandial GLP‐1 response (∆AUC), an sPLS regression analysis was performed (*n *= 324 pathways after filtering), identifying the super‐pathway of UDP‐glucose‐derived O‐antigen building blocks biosynthesis (PWY‐7328) as the most influential feature (Q^2^ total: 0.244). A subsequent correlation analysis revealed positive associations between PWY‐7328 and ∆AUC (*p =* 0.002, Figure 2C), as well as with differences in the postprandial GLP‐1 response at later time points (∆90 min to ∆240 min; all *p* < 0.05) (Table S3).

### 3.6 Impact of Microbial Evenness on Postprandial GLP‐1 Response

**FIGURE 3:**
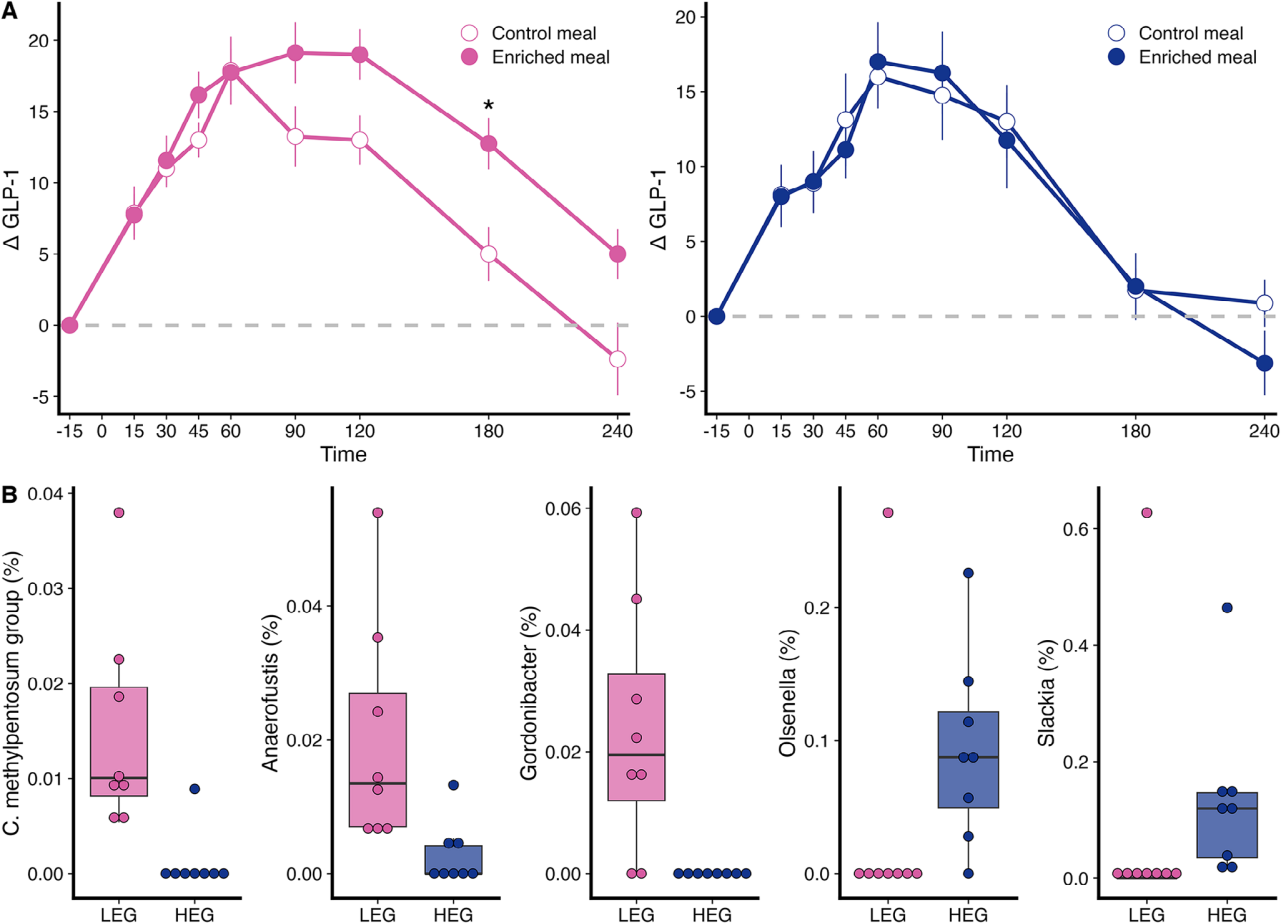
Differences between the low and high evenness groups in the postprandial GLP‐1 response and microbial composition. (A) Postprandial GLP‐1 response (pmol L^−1^) over 240 min in the LEG (pink) and HEG (blue), corrected for baseline values. Data are shown as means ± SEM. *p* values were derived from linear mixed models (* *p *< 0.05). (B) Relative abundances of significantly different genera between LEG and HEG identified using MaAsLin2 (*q* values < 0.1 corrected for multiple comparisons). *n* = 16. HEG, high evenness group; LEG, low evenness group.

To further explore how gut microbiota composition influences the meal‐induced GLP‐1 response, the participants were stratified into the LEG and HEG. Individuals in the LEG had a significantly stronger GLP‐1 response to EN compared to CON (*p = *0.012), whereas no difference between EN and CON was observed in the HEG (*p = *0.849) (Figure 3A). Additionally, in the LEG, a higher response to EN versus CON was detected at 180 min (*p = *0.042), with a trend at 240 min (*p* = 0.063).

Beyond the difference in Pielou's evenness (*p = *0.002), the LEG also had significantly lower Shannon entropy compared to the HEG (*p = *0.016) (Figure S2A, Table S4). Differences in microbial composition between the groups were visualized using principal coordinate analyses based on Bray–Curtis, Jaccard, and both unweighted and weighted UniFrac distances. PERMANOVA results indicated significant separation between LEG and HEG for Bray–Curtis (*p = *0.020), Jaccard (*p = *0.024), and weighted UniFrac (*p =* 0.043) distances (Figure S2B).

At the genus level, the abundances of the *Clostridium methylpentosum* group, *Anaerofustis*, and *Gordonibacter* were higher in the LEG compared to the HEG, whereas the abundances of *Olsenella* and *Slackia* were lower (all *q* < 0.1) (Figure 3B). Correlation analysis revealed that the abundance of the *C. methylpentosum* group was positively associated with ∆180 min GLP‐1 (*p =* 0.028) and ∆240 min GLP‐1 (*p =* 0.026), while the *Anaerofustis* abundance was positively associated with ∆240 min GLP‐1 (*p = *0.035) (Table S5).

### 3.7 Association Between Postprandial NEFA Response and Gut Microbiota

No significant associations were observed between differences in the meal‐induced NEFA response (∆AUC and ∆time points) and the calculated alpha diversity indices using linear regression analysis, except for a single inverse association with Observed features (∆120 min: *p *= 0.023; Table S6). Furthermore, no compelling evidence of a relationship between microbiome features and differences in NEFA response emerged from the sPLS regression models due to their low predictive ability (Q^2^ total: 0.070 for genera and −0.183 for pathways).

## 4 Discussion

This secondary analysis revealed an inverse association between alpha diversity and the postprandial GLP‐1 response (Figure 1), whereas postprandial NEFA responses appeared unrelated (Table S6). Notably, only individuals with lower microbial evenness exhibited higher postprandial GLP‐1 levels following EN compared to CON, with the most pronounced effect observed at 180 min after ingestion (Figure 3A). Genera such as the *C. methylpentosum* group, *E. ventriosum* group, and *Anaerofustis*—known producers of SCFAs—were identified as potentially important contributors to the pronounced GLP‐1 response (Figure 2A, Table S5). These findings support our hypothesis that baseline gut microbiota composition modulates the GLP‐1 response to a single meal fortified with *β*‐glucan‐rich oyster mushroom powder in adults with IGT.

It is well‐established that postprandial GLP‐1 secretion is induced by the interaction between nutrients in the chyme and enteroendocrine L‐cells, which are predominantly located in the ileum and colon, but also in the proximal jejunum [16, 17, 18, 19]. More recently, the gut microbiota has been shown to play a crucial role in GLP‐1 secretion throughout the gastrointestinal tract, particularly through the production of SCFAs, the main fermentation products of dietary fibers such as β‐glucans [20]. These microbial metabolites are produced primarily in the colon but also in the distal small intestine [21]. Animal studies have demonstrated that SCFAs stimulate GLP‐1 secretion by binding to free fatty acid receptor 2 (FFAR2; also known as GPR43) and FFAR3 (GPR41), which are located on the membranes of small intestinal and colonic L‐cells [22]. However, the precise localization of these receptors—whether on the apical or basolateral membranes of L‐cells—and their primary site of activation by SCFAs (luminal or vascular) remain poorly understood [22, 23]. Additionally, SCFAs may stimulate GLP‐1 secretion independently of these receptors by serving as an energy source [23]. Recent studies have also shown that SCFAs can activate intestinal gluconeogenesis [24] which might further contribute to GLP‐1 secretion. Therefore, interactions between L‐cells and microbial metabolites produced from the fermentation of *P. ostreatus* fiber may underlie the enhanced GLP‐1 response observed after EN compared to CON.

This assumption is supported by the observed positive association between the difference in meal‐induced GLP‐1 responses and the *E. ventriosum* group (Figure 2A), a potential SCFA‐producing genus previously shown to increase after 3 weeks of β‐glucan–chitin supplementation in individuals at cardiometabolic risk [25]. Chitin, which is also present in our mushroom powder (1.7 g per 20 g) [7], is a major component of fungal cell walls and forms covalent bonds with β‐glucans in *P. ostreatus* [26]. Therefore, the *E. ventriosum* group may have produced SCFAs by degrading both β‐glucans and chitin provided by EN, thereby stimulating postprandial GLP‐1 secretion.

Notably, the *C. methylpentosum* group, which was more abundant in the LEG and positively associated with the GLP‐1 response (Table S5), is closely related to *Paludicola psychrotolerans*, a strain known to utilize chitin as an energy source [27]. Therefore, the microbial capacity to degrade *P. ostreatus* fiber into SCFAs may be the underlying mechanism for the differential GLP‐1 responses observed in the LEG.

This is further supported by the higher abundance of *Anaerofustis* in the LEG (Figure 3B), another known SCFA producer [28]. Therefore, the greater specialization of the LEG microbiome in degrading fungal fiber into SCFAs, as compared to the HEG, may explain the enhanced GLP‐1 response following a single meal enriched with *P. ostreatus*.

A similar relationship between microbial specialization and alpha diversity has recently been observed when comparing vegans and omnivores: vegans had less diverse but more fiber‐fermenting microbiota, a trait associated with improved health outcomes [8]. Accordingly, the lower alpha diversity observed in participants with a greater GLP‐1 response to EN versus CON may reflect a more specialized, diet‐adapted microbiota rather than a dysbiotic gut environment.

Interestingly, the earliest differences in the meal‐induced GLP‐1 response and its associations with the gut microbiota were already visible 90 min postprandially (Table S3). This roughly corresponds to the previously reported gastric emptying lag‐time of 112 ± 5 min (mean ± SEM) observed in another study using the same enriched meal [29]. Therefore, we hypothesize that the observed differences in the GLP‐1 response were likely mediated by interactions with microbes in the small intestine rather than the colon.

This assumption appears plausible for many reasons. First, SCFA‐producing bacteria such as *Clostridium* species are present in the small intestine, suggesting that interactions between the *C. methylpentosum* group and nutrients from the test meal begin shortly after ingestion [30]. Second, the physiological response of enteroendocrine L‐cells to fluctuations in SCFA concentrations is believed to be more pronounced in the small intestine, given the consistently high SCFA levels in the colon [20]. Third, studies in mice reported that the gut microbiota has a stronger influence on the L‐cell transcriptome in the ileum compared to the colon [31].

Apart from SCFAs, amino acids in *P. ostreatus*, such as valine and phenylalanine, have been shown to stimulate GLP‐1 secretion [32, 33]. Moreover, several studies have demonstrated that the gut microbiota modulates the pool and bioavailability of amino acids by metabolizing them, interacting with proteolytic enzymes, and influencing gut permeability and amino acid absorption [34]. Therefore, differential modulation of the amino acid pool following *P. ostreatus* ingestion—depending on the gut microbiota composition—might contribute to the distinct GLP‐1 responses observed with EN. This effect may occur despite the relatively low amount of amino acids provided by the mushroom powder (2.1 g protein), which still represented a 30% increase compared to CON [7].

A strength of our study is its robust design (RCT, double‐blind, crossover) and the detailed, close‐meshed assessment of metabolic biomarkers alongside the characterization of the baseline fecal microbiome. This approach offers valuable insights into the role of gut microbes in the metabolic responses to a single meal. While the relatively small sample size may be considered a limitation—especially given the high inter‐individual variability expected in postprandial metabolic responses and gut microbiota composition— it was nevertheless sufficient to detect a significant association between the baseline microbiome and the GLP‐1 response induced by *P. ostreatus* fortification.

However, the use of fecal microbiome data as a proxy for the entire gut microbiota in human intervention studies presents limitations, as it provides limited information about microbial activity in the small intestine. This absence of direct data on microbial metabolites also restricts mechanistic insights. Therefore, larger studies are needed to fully elucidate the impact of the gut microbiome on GLP‐1 responses and to explore the underlying mechanisms. The use of smart capsules to collect samples from different regions of the gastrointestinal tract would be desirable.

Given the favorable postprandial effects observed after a single meal fortified with mushroom powder [7], RCTs investigating the effect of regular dietary fortification are of considerable interest. As sustained consumption of edible mushrooms such as *P. ostreatus* is likely to induce changes in the gut microbiome [35], future studies should also examine long‐term microbiome adaptations and their influence on GLP‐1 secretion.

In conclusion, our secondary analysis suggests that gut microbiota composition is an important modulator of the GLP‐1 response to a single meal fortified with β‐glucan‐rich oyster mushroom powder in adults with IGT. Therefore, the baseline gut microbiome may serve as a valuable tool for identifying individuals who are most likely to benefit from a given type and duration of a dietary intervention. Our findings provide further evidence of the individual interactions between diet and the gut microbiota, supporting the idea that microbiome characterization at baseline could enhance the design of nutritional intervention studies and inform personalized nutrition recommendations.

## Ethics Statement

The study protocol was approved by the ethics committees of the University of Bonn, Germany (project ID 167/18, approval date: 25/06/2018) and the Medical Association of North Rhine, Düsseldorf, Germany (project ID 2018254, approval date: 04/09/2018). All participants provided written informed consent, including consent for data publication, before enrollment.

## Conflicts of Interest

The authors declare no conflict of interest.

## Supporting information




**Supporting file 1**: mnfr70159‐sup‐0001‐SuppMat.pdf.

## Data Availability

The data presented in this publication will not be made available, as participants were assured in the informed consent form that personal data would not be disclosed to third parties.
