# Supplementary material for: GLP‐1 Responses to a Single Meal Fortified With Oyster Mushroom Powder in Adults With Impaired Glucose Tolerance Depend on the Gut Microbiota Composition Before the Meal
Source: Mol Nutr Food Res. 2025 Jun 29;69(20):e70159. doi: 10.1002/mnfr.70159 (PMC12538532; doi:10.1002/mnfr.70159)
Supplement: Supplementary file 1 — Supporting file 1: mnfr70159‐sup‐0001‐SuppMat.pdf. [file MNFR-69-e70159-s001.pdf]

SUPPORTING INFORMATION

GLP-1 Responses to a Single Meal Fortified With Oyster Mushroom Powder in Adults With Impaired Glucose Tolerance Depend on the Gut Microbiota Composition Before the Meal

Linda Klümpen, Anna Donkers, Waldemar Seel, Lisa Dicks, Jens Juul Holst, Peter Stehle, Marie-Christine Simon, Sabine Ellinger

Corresponding author: Prof. Dr. Sabine Ellinger. Institute of Nutritional and Food Science, Human Nutrition, University of Bonn, Käthe-Kümmel-Str. 1, 53115 Bonn, Germany. Email: ellinger@uni-bonn.de.

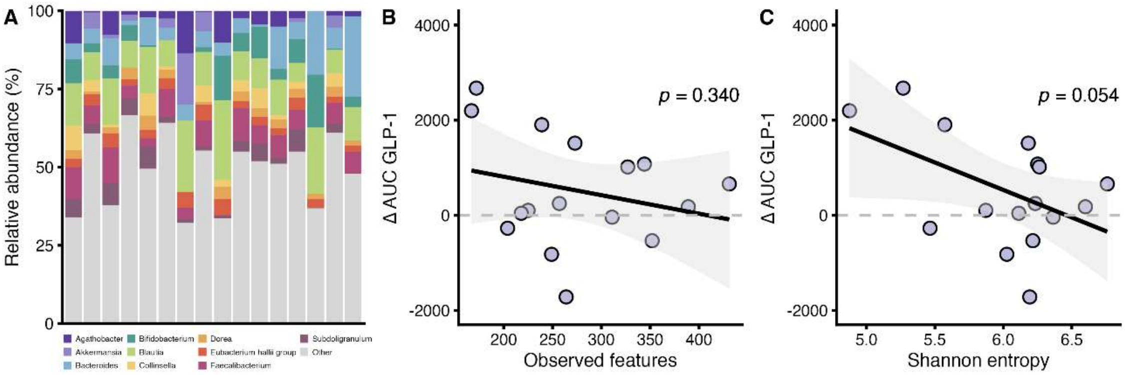

Supplementary Figure S1. Microbial composition at genus level and associations between the postprandial GLP-1 response and alpha diversity. (A) Relative abundance of the 10 most abundant genera of each participant. (B) Associations between differences in the meal-induced GLP-1 response (ΔAUC, pmol L^{-1} min) and Observed features. (C) Associations between differences in the meal-induced GLP-1 response (ΔAUC, pmol L^{-1} min) and Shannon entropy. *p* values were derived from linear regression analysis. * $p < 0.05$; ** $p < 0.01$. $n = 16$.

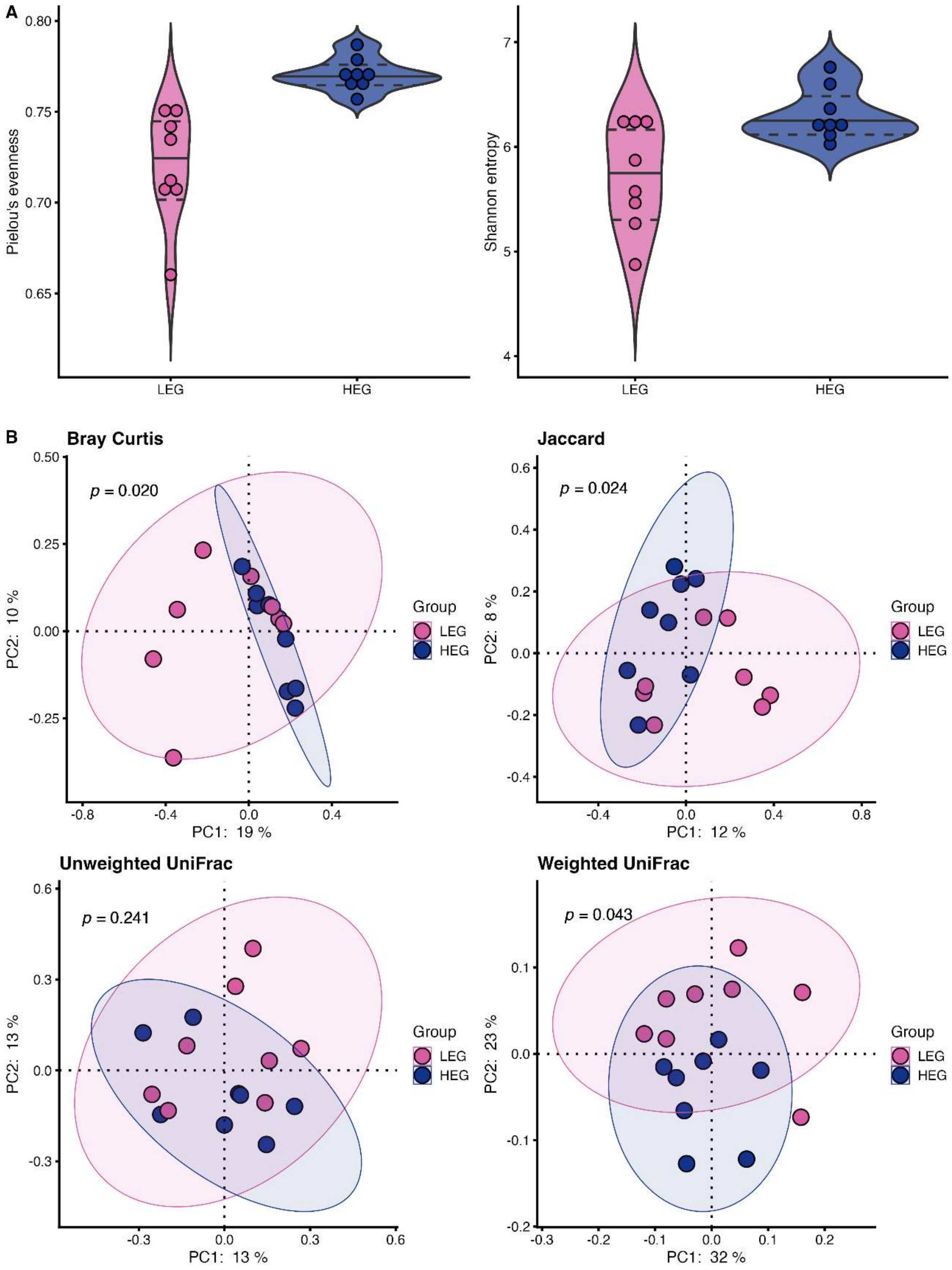

Supplementary Figure S2. Differences in alpha and beta diversity between LEG and HEG. (A) Pielou's evenness and Shannon entropy in the LEG and HEG. *p* values were derived from unpaired Student's *t*-test (* $p < 0.05$; ** $p < 0.01$). (B) PCoA plots showing the beta diversity based on Bray-Curtis dissimilarity, Jaccard distance, unweighted, and weighted UniFrac distance. *p* values were derived from PERMANOVA. $n = 16$. HEG, high evenness group; LEG, low evenness group.

Supplementary Table S1. Participant characteristics[a]

	Total (*n* = 16)	Females (*n* = 8)	Males (*n* = 8)
Age, y	34 (35)	31 (16)	53 (37)
BW, kg	100.8 ± 18.4	103.6 ± 17.3	98.0 ± 20.1
Fat mass, % BW	40.4 ± 8.9	47.6 ± 4.2	33.1 ± 5.5
Glucose, mg dL^{-1}	101 (23)	91 (18)	105 (46)
Insulin, mU L^{-1}	18.3 ± 9.5	18.5 ± 11.6	18.2 ± 7.6
HOMA-IR	4.7 ± 2.8	4.3 ± 3.0	5.2 ± 2.6
Triglycerides, mg dL^{-1}	155 ± 74	153 ± 62	157 ± 89
NEFAs, mg dL^{-1}	14.0 (10.7)	14.0 (12.3)	13.8 (9.9)
GLP-1, pmol L^{-1}	9.5 ± 6.2	7.9 ± 6.9	11.1 ± 5.4

[a]According to investigations in fasting state conducted at the first study day. Data are presented as means ± SD or as median (interquartile range) depending on data distribution. BW, body weight; GLP-1, glucagon-like peptide-1; HOMA-IR, homeostasis model assessment-insulin resistance; NEFAs, non-esterified free fatty acids.

Supplementary Table S2. Associations between alpha diversity of the gut microbiota and the differences in the baseline-corrected postprandial GLP-1 response between the enriched meal and the control meal

	Alpha diversity	β estimate	95% CI	*p* value	R^2
ΔAUC	Pielou's evenness	-0.573	-1.043, -0.103	0.020*	0.280
ΔAUC	Shannon entropy	-0.490	-0.990, 0.010	0.054	0.186
ΔAUC	Faith's PD	-0.563	-1.037, -0.089	0.023*	0.268
ΔAUC	Observed features	-0.255	-0.809, 0.299	0.340	-0.002
Δ90 min	Pielou's evenness	-0.323	-0.865, 0.220	0.223	0.040
Δ90 min	Shannon entropy	-0.281	-0.832, 0.269	0.291	0.013
Δ90 min	Faith's PD	-0.473	-0.978, 0.032	0.064	0.169
Δ90 min	Observed features	-0.161	-0.727, 0.404	0.550	-0.044
Δ120 min	Pielou's evenness	-0.544	-1.025, -0.062	0.030*	0.245
Δ120 min	Shannon entropy	-0.406	-0.930, 0.117	0.118	0.105
Δ120 min	Faith's PD	-0.462	-0.970, 0.046	0.072	0.157
Δ120 min	Observed features	-0.147	-0.714, 0.420	0.588	-0.048
Δ180 min	Pielou's evenness	-0.642	-1.081, -0.202	0.007**	0.370
Δ180 min	Shannon entropy	-0.557	-1.033, -0.081	0.025*	0.261
Δ180 min	Faith's PD	-0.427	-0.945, 0.092	0.099	0.124
Δ180 min	Observed features	-0.309	-0.854, 0.236	0.244	0.031
Δ240 min	Pielou's evenness	-0.647	-1.084, -0.209	0.007**	0.376
Δ240 min	Shannon entropy	-0.589	-1.052, -0.126	0.016*	0.301
Δ240 min	Faith's PD	-0.221	-0.780, 0.339	0.412	-0.019
Δ240 min	Observed features	-0.349	-0.887, 0.188	0.185	0.059

Scaled data are presented. *p* values derived from linear regression analysis (* < 0.05, ** < 0.01). *n* = 16. ΔAUC: baseline-corrected AUC. Δtimepoint: baseline-corrected concentrations at the single postprandial time points (i.e. changes versus baseline).

Supplementary Table S3. Associations between specific genera of the gut microbiota or microbial pathway and the differences in the baseline-corrected postprandial GLP-1 response

		r	***p* value**
Eubacterium ventriosum group	ΔAUC	0.7118	0.002**
	Δ90 min	0.7841	0.0003***
	Δ120 min	0.6043	0.0132*
	Δ180 min	0.7399	0.0011**
	Δ240 min	0.2851	0.2845
Ruminococcus gauvreauii group	ΔAUC	-0.688	0.0032**
	Δ90 min	-0.506	0.0458*
	Δ120 min	-0.709	0.0021*
	Δ180 min	-0.753	0.0008***
	Δ240 min	-0.699	0.0026**
Microbial pathway: PWY-7328	ΔAUC	0.7235	0.0015**
	Δ90 min	0.6794	0.0038**
	Δ120 min	0.6426	0.0073**
	Δ180 min	0.8445	3.87×10^{-5}***
	Δ240 min	0.5554	0.0255*

P values derived from Spearman's rank correlation analysis (* < 0.05, ** < 0.01, *** < 0.001). *n* = 16. ΔAUC: baseline-corrected AUC. Δtimepoint: baseline-corrected concentrations at the single postprandial time points (i.e. changes versus baseline).

Supplementary Table S4. Differences in alpha diversity of the gut microbiota between the low and high evenness groups

	**LEG (*n* = 8)**	**HEG (*n* = 8)**	**Δ**	**95% CI**	***p* value**
Pielou's evenness	0.72 ± 0.01	0.77 ± 0.00	-0.05	-0.08; -0.02	0.002**
Shannon entropy	5.72 ± 0.18	6.31 ± 0.09	-0.59	-1.04; -0.14	0.016*
Faith's PD	20.88 ± 2.07	22.39 ± 1.73	-1.51	-7.30; 4.28	0.585
Observed features	253.75 ± 27.02	299.00 ± 26.18	-45.25	-125.95; 35.45	0.249

Data are presented as means ± SEM. *p* values derived from unpaired Student's *t*-test (* < 0.05, ** < 0.01). HEG, high evenness group; LEG, low evenness group.

Supplementary Table S5. Associations between significantly different microbial genera between the low and high evenness groups and the differences in the baseline-corrected postprandial GLP-1 response

		r	***p* value**
Clostridium methylpentosum group	∆AUC	0.31	0.235
	∆90 min	0.25	0.346
	∆120 min	0.31	0.241
	∆180 min	0.55	0.028*
	∆240 min	0.56	0.026*
Anaerofustis	∆AUC	0.23	0.387
	∆90 min	0.05	0.845
	∆120 min	0.29	0.273
	∆180 min	0.27	0.304
	∆240 min	0.53	0.035*
Gordonibacter	∆AUC	0.31	0.244
	∆90 min	0.30	0.265
	∆120 min	0.16	0.552
	∆180 min	0.38	0.150
	∆240 min	0.32	0.226
Olsenella	∆AUC	-0.27	0.305
	∆90 min	-0.23	0.395
	∆120 min	-0.14	0.613
	∆180 min	-0.34	0.203
	∆240 min	-0.43	0.098
Slackia	∆AUC	-0.23	0.387
	∆90 min	-0.05	0.850
	∆120 min	-0.24	0.373
	∆180 min	-0.26	0.335
	∆240 min	-0.28	0.298

p values derived from Spearman's rank correlation analysis (* < 0.05). *n* = 16. ∆AUC: baseline-corrected AUC. ∆timepoint: baseline-corrected concentrations at the single postprandial time points (i.e. changes versus baseline).

Supplementary Table S6. Associations between alpha diversity of the gut microbiota and the baseline-corrected differences in the postprandial NEFA response between the enriched meal and the control meal

	**Alpha diversity**	**β estimate**	**95% CI**	***p* value**	**R^2**
ΔAUC	Pielou's evenness	0.083	-0.488, 0.654	0.760	-0.064
ΔAUC	Shannon entropy	0.317	-0.226, 0.861	0.231	0.037
ΔAUC	Faith's PD	0.369	-0.164, 0.902	0.159	0.075
ΔAUC	Observed features	0.457	-0.053, 0.967	0.075	0.153
Δ90 min	Pielou's evenness	0.086	-0.485, 0.657	0.750	-0.063
Δ90 min	Shannon entropy	-0.128	-0.696, 0.441	0.637	-0.054
Δ90 min	Faith's PD	-0.154	-0.720, 0.413	0.569	-0.046
Δ90 min	Observed features	-0.296	-0.843, 0.252	0.266	0.022
Δ120 min	Pielou's evenness	-0.199	-0.760, 0.363	0.461	-0.029
Δ120 min	Shannon entropy	-0.435	-0.951, 0.081	0.092	0.131
Δ120 min	Faith's PD	-0.476	-0.980, 0.029	0.063	0.171
Δ120 min	Observed features	-0.563	-1.037, -0.090	0.023*	0.269
Δ180 min	Pielou's evenness	-0.025	-0.598, 0.548	0.926	-0.071
Δ180 min	Shannon entropy	-0.208	-0.769, 0.353	0.439	-0.025
Δ180 min	Faith's PD	-0.357	-0.892, 0.179	0.175	0.065
Δ180 min	Observed features	-0.303	-0.850, 0.243	0.253	0.027
Δ240 min	Pielou's evenness	-0.099	-0.669, 0.472	0.716	0.682
Δ240 min	Shannon entropy	-0.247	-0.802, 0.309	0.357	-0.006
Δ240 min	Faith's PD	-0.119	-0.688, 0.450	0.660	-0.056
Δ240 min	Observed features	-0.301	-0.848, 0.245	0.257	0.026

Scaled data are presented. *p* values derived from linear regression analysis. *n* = 16. The NEFA concentration was analyzed in technical duplicates. ΔAUC: baseline-corrected AUC. Δtimepoint: baseline-corrected concentrations at the single postprandial time points (i.e. changes versus baseline).
